# Trisomy 8 presentation by inflammatory manifestations and its response to thalidomide: two case reports and narrative review

**DOI:** 10.3389/fped.2024.1431511

**Published:** 2024-08-20

**Authors:** Xiaohua Zhang, Yan Zhao, Yuting Pan, Jing Jin, Zhidan Fan, Haiguo Yu

**Affiliations:** ^1^ Department of Rheumatology and Immunology, Children’s Hospital of Nanjing Medical University, Nanjing, China; ^2^ Department of Ultrasonography, Children’s Hospital of Nanjing Medical University, Nanjing, China

**Keywords:** trisomy 8, Behcet’s disease, myelodysplastic syndrome, thalidomide, gastrointestinal involvement

## Abstract

**Objective:**

It has been recognized that there is a nexus among Trisomy 8 (T8), Behcet's disease (BD), and myelodysplastic syndrome (MDS). We reported a series of inflammatory features in 2 children with T8 without hematological involvement.

**Methods:**

2 children with trisomy 8 who were excluded from MDS were retrospectively collected from the Department of Rheumatology and Immunology, Children's Hospital of Nanjing Medical University, Nanjing.

**Results:**

Patients developed a range of inflammatory manifestations before a diagnosis of T8. The clinical manifestations of T8 patients vary from normal to severely disabled. Glucocorticoids and thalidomide can effectively relieve inflammation in patients with T8.

**Conclusion:**

The early clinical manifestations of T8 in children lack specificity, and the diagnosis is mainly based on karyotype analysis, gastrointestinal endoscopy and bone marrow aspiration findings. Active and effective immunoregulatory therapy and long-term follow-up can improve the prognosis of patients with T8.

## Introduction

T8 is a rare chromosomal abnormality disorder. T8 may be an acquired abnormality or a congenital abnormality that mainly occurs in the form of a chimera. However, complete T8 often results in miscarriage or embryo termination in the first trimester (1). T8, as an acquired condition, is found in haematological disorders, notably in myelodysplasia (MDS) and acute myeloid leukaemia (AML), and is restricted to the malignant cells. These arise in the bone marrow and may also be found in the peripheral blood (2). The phenotype of T8 varies from normal to severely disabled, characterized by mental retardation, facial deformities, skeletal deformities, delayed psychomotor development, urogenital and cardiovascular malformations, and developing neoplastic disorders (3). There is a strong link between MDS and T8 (+8-MDS) and autoinflammatory diseases, particularly Behcet's disease (BD) (4). Recent studies have focused on the autoimmune and autoinflammatory characteristics of T8-MDS while ignoring abnormal karyotypes without myelodysplastic abnormalities (5). BD is a chronic recurrent systemic inflammatory disease with unknown etiology that is mainly characterized by oral ulcers, genital ulcers, and ocular inflammation. Fever is a common manifestation of various diseases in children. In addition to infection-related causes, recurrent fever in children may also be caused by non-infective factors such as autoinflammation, thermic dysregulation, single gene mutations, and chromosome abnormalities. Relevant studies have shown that T8 is an important cause of inflammatory fever, which often causes periodic fever and Behcet's-like disease (5).

## Case presentation

### Case 1

**Figure 1:**
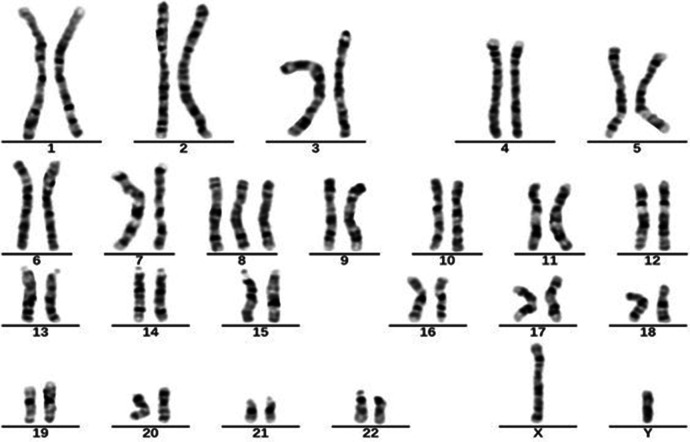
Karyotype of case 1: 47, XY+8[23]/46, XY[37].

**Figure 2:**
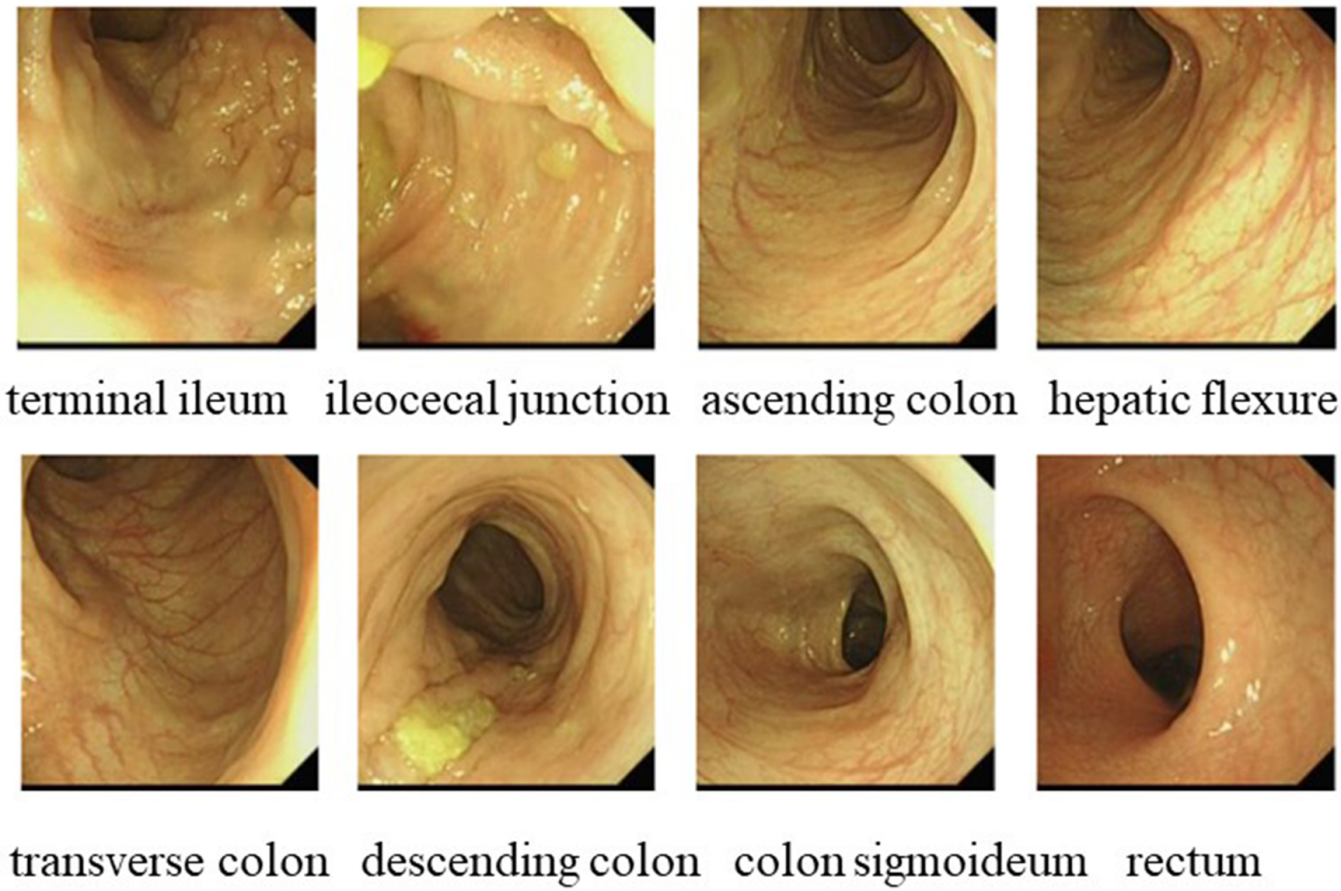
Gastrointestinal endoscopy in case 1 showed no abnormality.

In October 2021, a 4-year-old boy who presented to our hospital with recurrent fever and oral ulcers lasting for 6 months was suspected of having PFAPA syndrome due to recurrent regular fever (every month with a duration of 4–5 days), oral ulcers, tonsillitis, and elevated inflammatory markers, the absence of rash, and the ineffectiveness of antibiotic treatment. His C-reactive protein (CRP) (35.81 mg/L), erythrocyte sedimentation rate (ESR) (30 mm/h), procalcitonin (PCT) (1.57 ng/ml), and heparin-binding protein (85.5 ng/ml) levels were high. Etiological examinations, such as blood culture and urine culture, were both negative. The possibility of infection with Epstein–Barr virus, cytomegalovirus, hepatitis B virus, or Mycobacterium tuberculosis was excluded after pathogen antibody and DNA detection. IgA levels (1.51 g/L) were elevated, while IgM (0.799 g/L) levels were decreased. Tests for both autoinflammatory factors and anti-nuclear antibodies were negative. Fever was controlled in the patient treated with 17.5 mg of prednisone, an anti-inflammatory therapy. To determine the etiology, karyotype analysis was performed, and the patient was diagnosed with a duplication of chromosome 8 (Figure 1). In March 2023, the patient was readmitted for further investigation via gastrointestinal endoscopy (Figure 2) and bone marrow aspiration, both of which revealed no abnormalities. The child started receiving treatment with 25 mg of thalidomide once a night on March 9, 2023. Four months after the drug treatment, the patient's CRP (2.15 mg/L) and ESR (15 mm/h) were within the normal range. And during follow-up, the patient's symptoms were well controlled.

### Case 2

**Figure 3:**
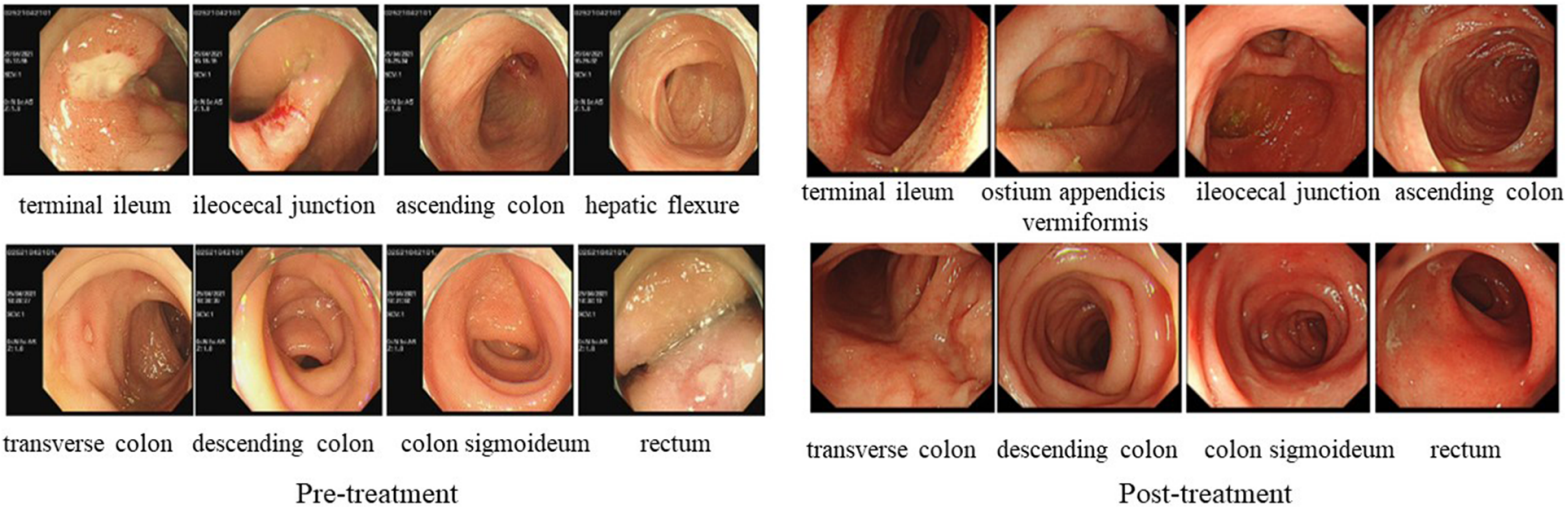
LEFT: gastrointestinal endoscopy in case 2 before treatment showed multiple ulcers throughout the distal ileum, ileocecal part, and colon. RIGHT: gastrointestinal endoscopy in case 2 after treatment showed multiple intestinal ulcers have recovered.

**Figure 4:**
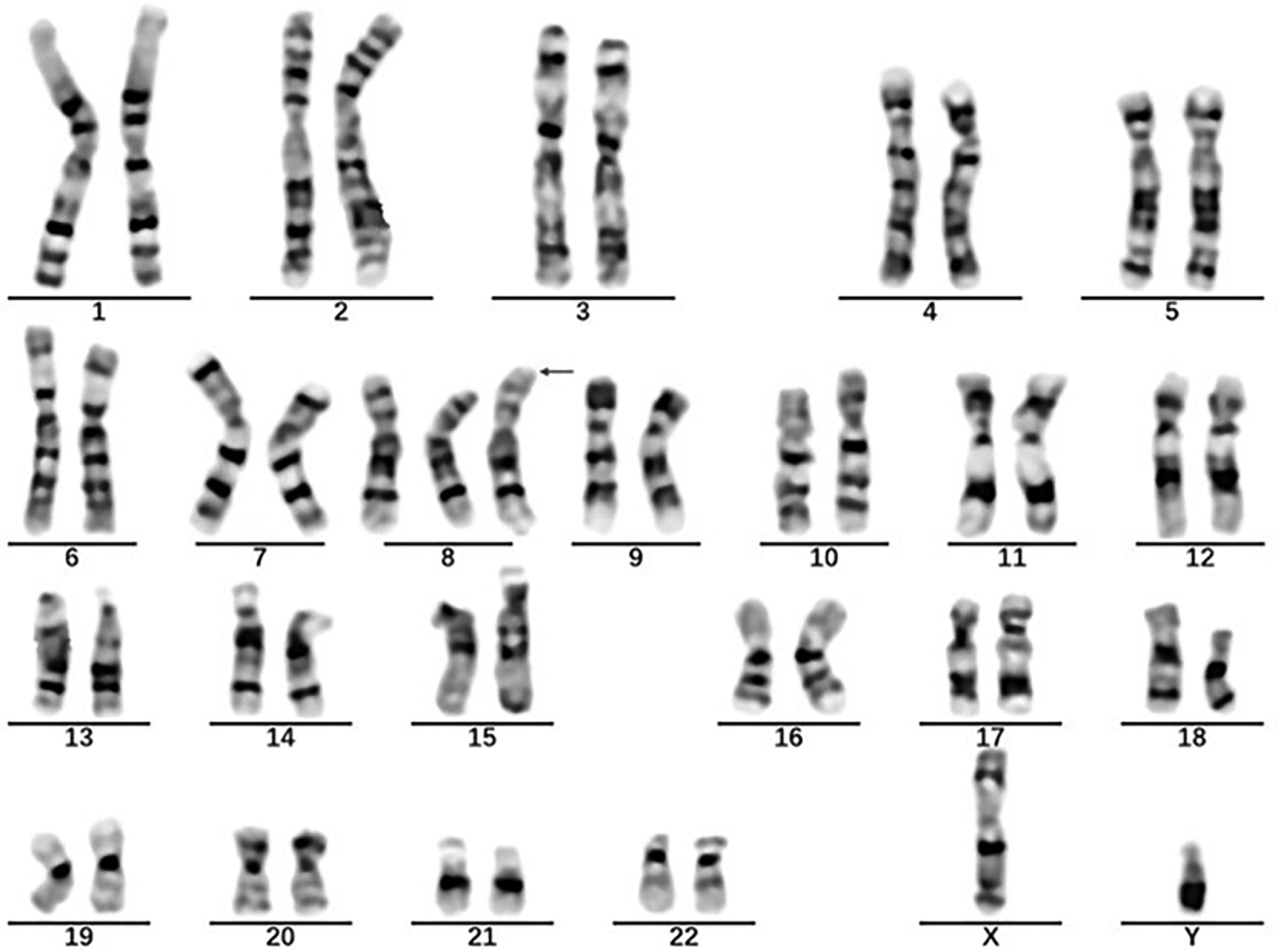
Karyotype of case 2: 47, XY+ 8[50]/46,XY[10].

In April 2021, a 7-year-old boy who presented to our hospital with “recurrent oral ulcers and fever for 5 years” and who was newly diagnosed with incomplete BD was diagnosed with fever and multiple refractory ulcers in the mouth, ileum, cecum, transverse colon, descending colon, and perianal region (Figure 3). There were no detectable skin lesions or ocular manifestations of BD, and the skin prick test was negative. The ESR (21 mm/h) and fecal calprotectin (197.7 ug/g) concentration were elevated. IgA (3.22 g/L) and IgG (13.1 g/L) levels were elevated, while IgM (0.755 g/L) levels were decreased. Tests for both autoinflammatory factors and anti-nuclear antibodies were negative. The patient was treated with a combination of mesalamine, sulfasalazine, and prednisone. In June 2021, chromosome karyotype analysis revealed a duplication on chromosome 8 and a mutation in the CYBB gene (Figure 4). Then, the patient underwent the first BM examination, which revealed that the morphology of bone marrow cells was normal. After 4 months and 2 years of drug treatment, the patient was re-examined via gastroenteroscopy, and no intestinal ulcers were observed in either examination (Figure 3). In addition, the patient's CRP (6.79 mg/L), ESR (8 mm/h), and ferritin (76.7 ng/ml) levels were all within normal limits upon re-examination. During the reduction of GCs, the patient's symptoms did not recur, and her blood cells did not decrease. Currently, the child is receiving oral administration of thalidomide for maintenance therapy and has undergone regular follow-up.

## Discussion and conclusion

Herein, we report two patients with T8 disease without myelodysplasia. These patients had autoinflammatory features, such as recurrent fever, rash, gastrointestinal involvement, and elevated inflammatory marker levels, without abnormalities in hematology, such as anemia and multilineage cytopenia. In Patient 1, periodic fever was the main manifestation. In Patient 2, the patient had gastrointestinal involvement. Studies of multiple patients have shown that almost all BD-like diseases occurring in +8-MDS patients are different from classical BD, with fewer eye lesions but more inflammation of the gut (mainly ileocecal). The BD-like manifestations of Patient 2 were consistent with this feature. Both of our patients experienced major symptom relief when treated with corticosteroids and thalidomide. In addition, corticosteroid reduction was successful, with no adverse events during follow-up.

T8 associated with Behçet's-like disease is less reported in children than in adults. We searched the literature on T8 with BD in children published in PubMed using keywords including “Trisomy 8” and “Behçet's disease”, and selected patients under the age of 18 with gastrointestinal involvement. In addition, we listed 2 cases with gastrointestinal involvement reported by ZHAO Wanwen et al. in a Chinese literature that is not indexed in PubMed in Table 1. We found a total of 9 children with T8 who have gastrointestinal manifestations, excluding our two patients. Almost all of these patients presented with gastrointestinal ulcers, of whom 4 patients had ileocecal involvement, and several patients had ulcers in the esophagus, upper digestive tract, ascending colon, transverse colon, descending colon, and sigmoid colon. Most patients had recurrent oral and genital ulcerations. However, skin lesions were present in only 2 of the 9 patients, and there were no eye lesions in any of the patients. Gastrointestinal involvement without eye lesions is one of the characteristic clinical findings in BD associated with T8. Our case 2 is consistent with this conclusion. In addition, all 9 patients had hematological system involvement, mainly manifested as AML and MDS, which occurred after the diagnosis of BD. In terms of treatment, glucocorticoids are used in more than ordinary cases and effectively alleviate the inflammatory manifestations in patients. Four patients were treated with tumor necrosis factor inhibitors (TNFi), thalidomide, and tacrolimus, which can effectively relieve gastrointestinal symptoms. Only 1 patient still experienced recurrent abdominal pain and intestinal ulcers after TNFi treatment, which were relieved after surgical treatment (6–10).

**Table 1:** Clinical features of 10 T8-BD patients with gastrointestinal involvement.

General condition	1	2	3	4	5	6	7	8	9	10
Gender	M	F	F	F	F	M	M	F	F	F
Age of onset	2	12	8	4	4	6	6	0	11	14
Age at diagnosis of BD	7	13	13	4	6	6	6	1.5	18	14
Complicated with hematologic malignancy	–	AML	MDS	MDS	AML	MDS	MDS	MDS	MDS	AML
Age at diagnosis of hematologic malignancies	–	14	13	4	7	6	6	3	18	15
Symptoms and signs										
Fever	Y	N	Y	Y	Y	Y	Y	Y	Y	Y
Oral ulcer	Y	Y	N	Y	Y	Y	Y	Y	Y	Y
Genital ulcer	Y	Y	N	N	N	N	N	N	Y	Y
Ocular symptom	N	N	N	N	N	N	N	N	N	N
Skin lesion	Y	N	N	Erythema nodosum	N	N	N	Ulcer of lower limb	N	N
Pathological changes and location of digestive tract	Ulcer, between the end of the ileum and the descending colon, Perianal region	Ulcer, ileocecal junction	Inflammation, the left colon	Ulcer, transverse and ascending colon	Ulcer, between the ileocecal part and the sigmoid colon	Ulcer, upper gastrointestinal tract	Ulcer, esophagus	Ulcer, upper gastrointestinal tract	Ulcer, cecum	Ulcer, ileocecal junction
Accessory examination										
Hemocyte	N	N	Y	Y	Y	Y	Y	Y	N	N
Inflammatory index	ESR, FCAL	CRP, ESR	CRP, ESR	CRP	–	–	–	–	CRP	CRP, ESR
Immune globulin	IgA, IgG↑, IgM↓	N	N	N	–	–	–	–	N	–
Alexin	N	N	N	–	–	–	–	–	–	–
Antinuclear antibodies	N	Granular type, cytoplasmic granular type	N	N	–	–	–	–	N	N
Treatment and prognosis										
Treatment	GC, SAL	GC, TNFi, MTX, CT	GC, CsA	HSCT	Tac, MTX, CsA, SAL, HSCT, CT	GC, HSCT	GC, TOZ, HSCT	GC, HSCT	TNFi, MTX, HSCT	GC, TNFi, MTX, CT
Dead or not	N	Y	N	N	Y	N	Y	N	N	Y

**Figure 5:**
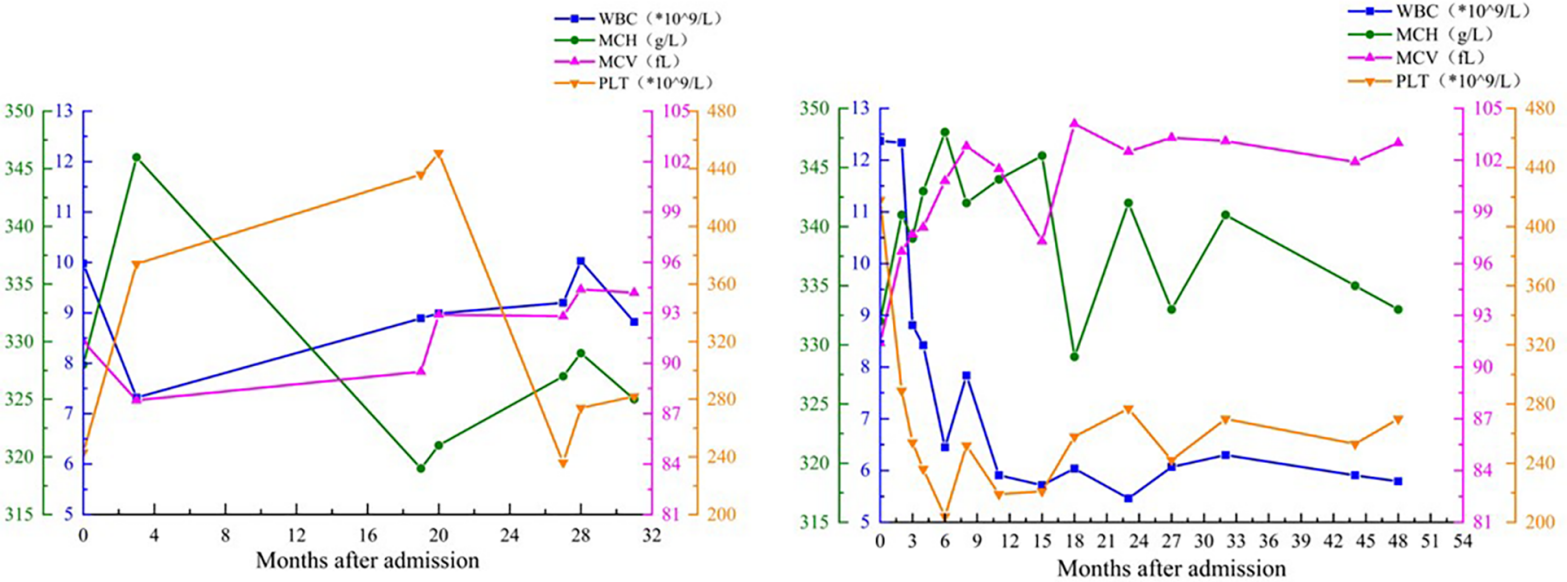
Peripheral blood cells parameters of 2 patients after admission.

In cases of cytogenetic abnormalities, T8 is the only chromosomal aberration in 5% of AML patients and 10% of MDS patients (11). However, in the absence of morphological features to diagnose MDS, T8 is not sufficient to diagnose MDS; in other words, T8 alone is not sufficient to induce the occurrence of hematological diseases. Significantly, patients with T8 disease are at increased risk of developing bone marrow malignancies, and patients with T8 disease without MDS may develop MDS over time (4). Whether and how long children's disease progresses to MDS is unknown and may vary depending on factors such as disease severity and treatment response. Therefore, for these two T8 patients, continuous follow-up of the blood system is highly important, even though no significant abnormalities have been observed in the peripheral blood in these two children at present (Figure 5).

Chromosome 8 contains genes that regulate immune and inflammatory responses, and abnormal chromosome expression causes elevated levels of interleukin (IL)-1β, IL-6, and other inflammatory factors. Repeatedly high levels of inflammatory factors can activate neutrophils and promote their production of reactive oxygen species, leading to the occurrence and development of BD (1). However, only a minority of T8 patients exhibit BD-like disease, suggesting that T8 is also insufficient to cause autoinflammatory or BD-like phenotype (11). A retrospective analysis of MDS patients and BD patients revealed that the occurrence of intestinal ulcers was closely related to MDS and T8, while fever seemed to be mainly related to T8 (12). Furthermore, in previous reports, T8 was the most frequently detected chromosomal abnormality in cases of inflammatory fever, and it often leads to periodic fever in children (4). Therefore, for children with BD and unexplained fever, the possibility of T8 should be considered when there are atypical manifestations, such as developmental abnormalities, special facial features, joint malformations, atypical endoscopic intestinal lesions, or signs of hematological disease (1). A thorough physical examination revealed that patient 1 had finger deformities (claw hands), a spherical nose, and a slightly wider cleft palate. Patient 2 had an occult penis. Therefore, at the first admission of these two patients, we performed a chromosome karyotype examination for each patient and detected +8 as early as possible, which is highly important for disease control and prognosis in children.

At present, there are no guidelines for the diagnosis and treatment of T8 children with BD, fever, or other related inflammatory manifestations, and it is necessary to develop an individualized treatment plan according to clinical symptoms and immunological phenotypes. Glucocorticoids are widely used in T8 patients and can effectively control T8-related symptoms, such as fever, abdominal pain, and oral ulcers, and inflammation indicators are significantly reduced. However, the side effects of glucocorticoids cannot be ignored, especially for growing children. Timely and safe reduction of glucocorticoids is essential for alleviating side effects, but adverse reactions such as fever and elevated inflammatory indicators may occur during the process of reduction (4), which is also a difficult challenge in the treatment process. Therefore, the use of immunosuppressants and biologics is crucial for promoting glucocorticoid reduction and improving systemic inflammation. These two patients and five nonhematological T8 children in the study by Zhao et al. had a good prognosis and no deaths after aggressive treatment. However, if the blood system of a child with T8 disease is affected, the patient may not respond to conventional drug therapy, which may lead to adverse outcomes, and early bone marrow or peripheral blood stem cell transplantation may be able to completely control the disease (1).

Thalidomide (THD) is an immunomodulatory drug broadly applied in BD treatment. A large number of clinical cases have demonstrated that THD can rapidly improve oral and genital lesions in patients with Behcet's disease, prolong the recurrence time, and reduce the severity of symptoms (13). TNF-α is a new cytopathogenic factor of BD and MDS that can induce programmed cell death by inhibiting normal hematopoietic cells and subsequently accelerating the disease progression of BD or MDS (14). Thalidomide exerts antivascular effects by inhibiting the secretion of TNF-α by white blood cells and reducing the production of vascular endothelial growth factor (VEGF). In addition, thalidomide is more affordable than other biologics (14). Patients with gastrointestinal involvement of Behçet's syndrome who were refractory to the conventional therapy can often get clinical and endoscopic remission with TNF-alpha antagonists and/or thalidomide (15). Based on clinical experience and literature reports, THD was also used in our 2 patients, and satisfactory results were obtained.

However, our study is only a single-center report from our institution and not a large multicenter study; thus, the results are merely a preliminary summary of the clinical characteristics of such patients, and the specific mechanisms require further investigation. Additionally, there is currently a lack of reference guidelines for the treatment and clinical management of T8-BD children, particularly regarding high inflammatory manifestations and gastrointestinal involvement. Besides, the risk of future hematological disorders in these children is unknown.

In conclusion, children with T8 can have a wide variety of phenotypes that vary in severity. We focus on the fact that the early clinical manifestations of T8 in children lack specificity. Therefore, for children with BD, fever of unknown origin, recurrent oral ulcers, and unexplained gastrointestinal ulcers, perfecting chromosome karyotype analysis, gastrointestinal endoscopy, and bone marrow aspiration as soon as possible is highly important for the diagnosis, treatment, and prognosis of patients. Both of our cases treated with THD achieved favorable therapeutic effects, which may provide clinical physicians with treatment experience for T8 associated with gastrointestinal involvement. Active and effective immunoregulatory therapy and long-term follow-up can improve the prognosis of patients with T8.

## Data Availability

The original contributions presented in the study are included in the article/Supplementary Material, further inquiries can be directed to the corresponding authors.
